# Private genome analysis through homomorphic encryption

**DOI:** 10.1186/1472-6947-15-S5-S3

**Published:** 2015-12-21

**Authors:** Miran Kim, Kristin Lauter

**Affiliations:** 1 Department of Mathematical Sciences, GwanAkRo 1, Seoul, Korea; 2 Cryptography Research Group, Microsoft Research, Redmond, WA, USA

**Keywords:** Homomorphic encryption, Genome-wide association studies, Hamming distance, Approximate Edit distance

## Abstract

**Background:**

The rapid development of genome sequencing technology allows researchers to access large genome datasets. However, outsourcing the data processing o the cloud poses high risks for personal privacy. The aim of this paper is to give a practical solution for this problem using homomorphic encryption. In our approach, all the computations can be performed in an untrusted cloud without requiring the decryption key or any interaction with the data owner, which preserves the privacy of genome data.

**Methods:**

We present evaluation algorithms for secure computation of the minor allele frequencies and *χ*^2 ^statistic in a genome-wide association studies setting. We also describe how to privately compute the Hamming distance and approximate Edit distance between encrypted DNA sequences. Finally, we compare performance details of using two practical homomorphic encryption schemes - the BGV scheme by Gentry, Halevi and Smart and the YASHE scheme by Bos, Lauter, Loftus and Naehrig.

**Results:**

The approach with the YASHE scheme analyzes data from 400 people within about 2 seconds and picks a variant associated with disease from 311 spots. For another task, using the BGV scheme, it took about 65 seconds to securely compute the approximate Edit distance for DNA sequences of size 5K and figure out the differences between them.

**Conclusions:**

The performance numbers for BGV are better than YASHE when homomorphically evaluating deep circuits (like the Hamming distance algorithm or approximate Edit distance algorithm). On the other hand, it is more efficient to use the YASHE scheme for a low-degree computation, such as minor allele frequencies or *χ*^2 ^test statistic in a case-control study.

## Introduction

The rapid development of genome sequencing technology has led to the genome era. We expect that the price of a whole genome sequence will soon be $1K in a day, which enables researchers to access large genome datasets. Moreover, many genome projects like the Personal Genome Project (PGP) [1] and the HapMap Project [2] display genotypic information in public databases, so genomic data has become publicly accessible.

While genome data can be used for a wide range of applications including healthcare, biomedical research, and forensics, it can be misused, violating personal privacy via genetic disease disclosure or genetic discrimination. Even when explicit identifiers (*e.g.*, name, date of birth or address) are removed from genomic data, one can often recover the identity information [3-5]. For these reasons, genomic data should be handled with care.

There have been many attempts to protect genomic privacy using cryptographic methods. In particular, it has been suggested that we can preserve privacy through (partially) homomorphic encryption, which allows computations to be carried out on ciphertexts. Kantarcioglu et al. [6] presented a novel framework that allows organizations to support data mining without violating genomic privacy. Baldi et al. [7] proposed a cryptographic protocol to determine whether there exists a biological parent-child relationship between two individuals. Ayday et al. [8] recently conducted privacy-preserving computation of disease risk based on genomic and non-genomic data. However, these methods used homomorphic computation involving a single operation on ciphertexts (*e.g.*, either additions or multiplications, not both), thus they could support a limited set of genomic queries.

Fully homomorphic encryption (*e.g.*, [9-11]) permits encrypted data to be computed on without decryption, so it allows us to evaluate arbitrary arithmetic circuits over encrypted data. Thus, we can privately perform all types of genome analysis using Homomorphic Encryption (HE) cryptosystems. Moreover, we can delegate intensive computation to a public cloud and store large amounts of data in it.

Recently, many protocols to conduct privacy-preserving computation of genomic tests with fully homomorphic encryption have been introduced. Yasuda et al. [12] gave a practical solution for computation of multiple Hamming distance values using the LNV scheme [13] on encrypted data, so to find the locations where a pattern occurs in a text. Graepel et al. [14] and Bos et al. [15] applied HE to machine learning, and described how to privately conduct predictive analysis based on an encrypted learned model. Lauter et al. [16] gave a solution to privately compute the basic genomic algorithms used in genetic association studies. Cheon et al. [17] described how to calculate edit distance on homomorphically encrypted data.

In this paper, we propose efficient evaluation algorithms to compute genomic tests on encrypted data. We first consider the basic tests which are used in Genome-Wide Association Studies (GWAS). They are conducted to analyze the statistical associations between common genetic variants in different individuals. In particular, we focus on the minor allele frequencies (MAFs) and *χ*^2 ^test statistic between the variants of case and control groups. Secondly, we consider DNA sequence comparison which can be used in sequence alignment and gene finding. We show how to privately compute the Hamming distance and approximate Edit distance on encrypted data. We also adapt these methods to the practical HE schemes *− *BGV scheme [18] by Gentry, Halevi and Smart and YASHE scheme [19] by Bos, Lauter, Loftus and Naehrig. Finally, we compare the performance of the two encryption schemes in these contexts. In practice, we take advantage of batching techniques to parallelize both space and computation time together.

One possible scenario could be of interest in situations involving patients, a data owner (*e.g.*, a healthcare organization or a medical center) and a public cloud. In our solution, a data owner wants to store large amounts of data in the cloud and many users may interact with the same data over time. The cloud can handle all that interaction through computation on encrypted data, so it does not require further interaction from the data owner. The patients can upload their encrypted data directly to the cloud using the public key. The genomic tests are performed on the cloud and the encrypted results are returned to the data owner. Finally, the data owner decrypts the results using the secret key to share it with the patient. All the computations in the cloud are performed on encrypted data without requiring the decryption key, so the privacy of genomic data can be protected by the semantic security of the underlying HE schemes.

## Background

The iDASH (Integrating Data for Analysis, 'anonymization' and SHaring) National Center organized the iDASH Privacy & Security challenge for secure genome analysis. This paper is based on a submission to the iDASH challenge which consisted of two tasks: i) secure outsourcing of GWAS and ii) secure comparison between genomic data.

### Two tasks for iDASH challenge

Given the encrypted genotypes of two groups of individuals over many single nucleotide variants (SNVs), the goal of the first task is to privately compute the MAFs in each group and a *χ*^2 ^test statistic between the two groups on each site.

Suppose that *A *and *B *are two alleles of the gene, and let *n_AA_*, *n_AB_*, *n_BB _*denote the numbers of observed individuals for genotypes *AA, AB, BB*, respectively. The allele counts of *A *and *B *are given by $n_{A}\overset{let}{=}2n_{AA}+n_{AB}$ and $n_{B}\overset{let}{=}2n_{BB}+n_{AB}$. Then the MAF of the given alleles is defined by

$$\frac{\text{min}\left(n_{A},n_{B}\right)}{n_{A}+n_{B}}.$$

If we let *N *be the total number of people in a sample population, the total number of alleles in the sample is *n_A _*+ *n_B _*= 2*N*, so we compute only one of two allele counts in encrypted form. The minimum can then easily be computed after decryption and we obtain the MAF by one division by 2*N *.

The *χ*^2 ^test statistic in case-control groups is computed based on the allelic contingency table (Table 1):

**Table 1:** Allelic Contingency Table

	Allele type		Total
		
	*A*	*B*	
Case	*n_A_*	*n_B_*	*R *= 2*N*
Control	$n_{A}^{\prime }$	$n_{B}^{\prime }$	*S *= 2*N*
Total	$G=n_{A}+n_{A}^{\prime }$	$K=n_{B}+n_{B}^{\prime }$	*T *= 4*N*

$$\frac{T\left(n_{A}{n^{\prime }}_{B}-n_{B}{n^{\prime }}_{A}\right)}{R\cdot S\cdot G\cdot K}.$$

**Algorithm 1 **Hamming Distance Algorithm

1: *h ← *0

2: **for ***i *∈  L**do**

3:   **if **('*x_i_*.sv' or '*y_i_*.sv') in {'INS', 'DEL'} **then**

4:      *h_i _← *0

5:   **else if **((*x_i _*or *y_i_*) == '∅') or

6:   ((*x_i_*.ref == *y_i_*.ref) and (*x_i_*.alt ! = *y_i_*.alt)) **then**

7:      *h_i _← *1

8:   **else**

9:      *h_i _← *0

10:   **end if**

11:   *h ← h *+ *h_i_*

12: **end for**

13: **return ***h*

We observe that the test can be written as a function of *n*_*A *_and $n_{A}^{\prime }$. More precisely, it is expressed as

$$\begin{array}{c} \frac{4N{\left(n_{A}\left(2N-{n^{\prime }}_{A}\right)-{n^{\prime }}_{A}\left(2N-n_{A}\right)\right)}^{2}}{2N\cdot 2N\cdot G\cdot K}\\ =\frac{4N{\left(n_{A}-{n^{\prime }}_{A}\right)}^{2}}{\left(n_{A}+{n^{\prime }}_{A}\right)\cdot \left(4N-\left(n_{A}+{n^{\prime }}_{A}\right)\right)}. \end{array}$$

Let $n_{A}^{\left(j\right)}$ and ${n^{\prime }}_{A}^{\left(j\right)}$ denote the allele counts of A at SNV *j *in the case group and control group, respectively. As discussed above, it suffices to compute $\left(n_{A}^{\left(j\right)}+{n^{\prime }}_{A}^{\left(j\right)}\right)$ and $\left(n_{A}^{\left(j\right)}-{n^{\prime }}_{A}^{\left(j\right)}\right)$ over encrypted data.

The goal of the second task is to privately compute the Hamming distance and approximate Edit distance between the encrypted genome sequences. Suppose that two participants have Variation Call Format (VCF) files which summarize their variants compared with the reference genome (*e.g.*, insertion, deletion, or substitution at a given position of a given chromosome). If there is only one record in the VCF files at a specified location, the other one is considered to be an empty set ('∅'). Let  L be a list indexed by the positions of two participants. Then we can define the Hamming distance as described in Algorithm 1, where "*x_i_*.sv" denotes the type of structural variant relative to the reference, "*x_i_*.ref " the reference bases and "*x_i_*.alt" the alternate non-reference alleles.

The standard dynamic programming approach to compute the full Wagner-Fischer Edit distance [20] is computed in a recursive way, so the multiplicative depth of the circuit to be homomorphically evaluated is too large. Recently, Cheon et al. [17] presented an algorithm to compute the WF Edit distance over packed ciphertexts but it took about 27 seconds even on length 8 DNA sequences. On the other hand, in this task we are given the distance to a public human DNA sequence (called the reference genome), which allows us to efficiently approximate the Edit distance using Algorithm 2. It is calculated based on the set difference metric, which enables parallel processing in computation.

**Algorithm 2 **Approximate Edit Distance Algorithm

1: *e ← *0

2: **for **i∈L**do**

3:   **if ***x_i _*== '∅' **then**

4:      *D*(*x_i_*) *← *0

5:   **else if **'*x_i_*.sv' == 'DEL' **then**

6:      *D*(*x_i_*) *← *len(*x_i_*.ref)

7:   **else**

8:      *D*(*x_i_*) *← *len(*x_i_*.alt)

9:   **end if**

10:   Define *D*(*y_i_*) with the same way as *D*(*x_i_*)

11:   **if **((*x_i_*.ref == *y_i_*.ref) and (*x_i_*.alt == *y_i_*.alt)) **then**

12:      *e_i _← *0

13:   **else**

14:      *e_i _← *max{*D*(*x_i_*), *D*(*y_i_*)}

15:   **end if**

16:   *e ← e *+ *e_i_*

17: **end for**

18: **return ***e*

### Practical homomorphic encryption

Fully Homomorphic cryptosystems allow us to homomorphically evaluate any arithmetic circuit without decryption. However, the noise of the resulting ciphertext grows during homomorphic evaluations, slightly with addition but substantially with multiplication. For efficiency reasons for tasks which are known in advance, we use a more practical *Somewhat Homomorphic Encryption *(SHE) scheme, which evaluates functions up to a certain complexity. In particular, two techniques are used for noise management of SHE: one is the *modulus-switching *technique introduced by Brakerski, Gentry and Vaikuntanathan [21], which scales down a ciphertext during every multiplication operation and reduces the noise by its scaling factor. The other is a *scale-invariant *technique proposed by Brakerski [22] such that the same modulus is used throughout the evaluation process.

Let us denote by [·]*_q _*the reduction modulo *q *into the interval (-q/2,q/2]∩ℤ of the integer or integer polynomial (coefficient-wise). For a security parameter *λ*, we choose an integer *m *= *m*(*λ*) that defines the *m*-th cyclotomic polynomial Φ*_m_*(*x*). For a polynomial ring $R=\mathbb{Z}\left[x\right]/\left(\Phi_{m}\left(x\right)\right)$, set the plaintext space to *R_t _*:= *R/tR *for some fixed *t ≥ *2 and the ciphertext space to *R_q _*:= *R/qR *for an integer *q *= *q*(*λ*). Let *χ *= *χ*(*λ*) denote a noise distribution over the ring *R*. We use the standard notation a←D to denote that *a *is chosen from the distribution  D. Now, we recall the BGV scheme [18] and the scale-invariant YASHE scheme [19].

#### The BGV scheme

Gentry, Halevi and Smart [18] constructed an efficient BGV-type SHE scheme. The security of this scheme is based on the (decisional) Ring Learning With Errors (RLWE) assumption, which was first introduced by Lyubashevsky, Peikert and Regev [23]. The assumption is that it is infeasible to distinguish the following two distributions. The first distribution consists of pairs (*a_i_, u_i_*), where *a_i_, u_i _← R_q _*uniformly at random. The second distribution consists of pairs of the form (*a_i_, b_i_*) = (*a_i_, a_i_*s + *e_i_*) where *a_i _← R_q _*drawn uniformly and s, *e_i _← χ *. Note that we can generate RLWE samples as (*a_i_, a_i_*s+*te_i_*) where *t *and *q *are relatively prime. To improve efficiency for HE, they use very sparse secret keys s with coefficients sampled from {*−*1, 0, 1}.

Here is the SHE scheme of [18]:

• ParamsGen: Given the security parameter *λ*, choose an odd integer *m*, a chain of moduli *q*_0 _<*q*_1 _< ⋯ <*q*_*L*−1 _= *q*, a plaintext modulus *t *with 1 <*t *<*q*_0_, and discrete Gaussian distribution *χ_err _*. Output (*m*, {*q_i_*}, *t, χ_err_*).

• KeyGen: On the input parameters, choose a random s from {0, ± 1}^*φ*(*m*) ^and generate an RLWE instance (*a, b*) = (*a*, [*a*s + *te*]*_q _*) for *e ← χ_err_*. We set the key pair: (pk, sk) = ((*a, b*), s) with an evaluation key $evk\in R_{P\cdot q_{L-2}}^{2}$ for a large integer *P*.

• Encryption: To encrypt *m *∈ *R_t_*, choose a small polynomial *v *and two Gaussian polynomials *e*_0_, *e*_1 _over *Rq *. Then compute the ciphertext given by Enc(*m*, pk) = (*c*_0_, *c*_1_) = (*m*, 0) + (*bv *+ *te*0, *av *+*te*_1_) ∈ $R_{q}^{2}$.

• Decryption: Given a ciphertext ct = (*c*_0_, *c*_1_) at level *l*, output $\text{Dec(ct,sk)}={\left[c_{0}-s\cdot c_{1}\right]}_{q_{l}}$ mod *t *where the polynomial ${\left[c_{0}-s\cdot c_{1}\right]}_{q_{l}}$ is called the *noise *in the ciphertext ct.

• Homomorphic Evaluation: Given two ciphertexts ct = (*c*_0_, *c*_1_) and $\text{ct'}=\left(c_{0}^{\prime },c_{1}^{\prime }\right)$ at level *l*, the homomorphic addition is computed by $\text{c}{\text{t}}_{\text{add}}=\left({\left[c_{0}+c_{0}^{\prime }\right]}_{q_{l}},{\left[c_{1}+c_{1}^{\prime }\right]}_{q_{l}}\right)$. The homomorphic multiplication is computed by ct_mult _= SwitchKey(*c*_0 _∗ *c*_1_ , evk) where $c_{0}*c_{1}=\left({\left[c_{0}c_{0}^{\prime }\right]}_{q_{l}},{\left[c_{0}c_{1}^{\prime }+c_{1}c_{0}^{\prime }\right]}_{q_{l}},{\left[c_{1}c_{1}^{\prime }\right]}_{q_{l}}\right)$ and the key switching function SwitchKey is used to reduce the size of ciphertexts to two ring elements. We also apply modulus switching from *q_i _*to *q*_*i*−1 _in order to reduce the noise. If we reach the smallest modulus *q*_0_, we can no longer compute on ciphertexts.

Smart and Vercauteren [24] observed that *R_t _*is isomorphic to $\prod_{i=1}^{\ell }{\mathbb{Z}}_{t}\left[x\right]/f_{i}\left(x\right)$ if Φ*_m_*(*x*) factors modulo *t *into *ℓ *irreducible factors *f_i_*(*x*) of the same degree. Namely, a plaintext polynomial *m *can be considered as a vector of *ℓ *small polynomials, *m *mod *f_i_*, called *plaintext slots*. We can also transform the plain-text vector $\left(m_{1},\ldots ,m_{r}\right)\in \prod_{i=1}^{\ell }{\mathbb{Z}}_{t}\left[x\right]/f_{i}\left(x\right)$ to an element *m *∈ *R_t _*using the polynomial Chinese Remainder Theorem (*i.e., m *= CRT(*m*_1_, ..., *m_r_*)). In particular, it is possible to add and multiply on the slots: if *m, m′ *∈ *R_t _*encode (*m*_1_ , ..., *m_ℓ_*) and $\left(m_{1}^{\prime },\ldots ,m_{\ell }^{\prime }\right)$ respectively, then we see that $m+m^{\prime }=m_{i}+m_{i}^{\prime }$ mod *f_i _*and $m\cdot m^{\prime }=m_{i}\cdot m_{i}^{\prime }$ mod *f_i_*. This technique was adapted to the BGV scheme.

#### The YASHE scheme

A practical SHE scheme, YASHE, was proposed in [19] based on combining ideas from [22,25,26]. The security of this scheme is based on the hardness of the RLWE assumption similar to the one for BGV. It also relies on the Decisional Small Polynomial Ratio (DSPR) assumption which was introduced by Lopez-Alt, Tromer, and Vaikuntanathan [26]. Let $t\in R_{q}^{\times }$ be invertible in *R_q_*, *y_i _*∈ *R_q _*and *z_i _*= *y_i_/t *( mod *q*) for i = 1, 2. For *z *∈ *R_q_*, and, we define *χ_z _*= *χ *+ *z *to be the distribution shifted by *z*. The assumption is that it is hard to distinguish elements of the form *h *= *a/b*, where *a ← y*_1 _+ *tχ_z_, b ← y*_2 _+ *tχ_z_*, from elements drawn uniformly from *R_q _*. The YASHE scheme consists of the following algorithms.

• ParamsGen: Given the security parameter *λ*, choose *m *to be a power of 2 (the *m*-th cyclotomic polynomial is Φ*_m_*(*x*) = *x^n ^*+ 1 (*n *= *φ*(*m*) = *m/*2), modulus *q *and *t *with 1 *< t < q*, truncated discrete Gaussian distribution *χ_err _*on *R *such that the coefficients of the polynomial are selected in the range [*−B*(*λ*), *B*(*λ*)]), and an integer base *ω >*1. Output (*m, q, t, χ_err_, ω*).

• KeyGen: On the input parameters, sample *f′, g ← *{0, ± 1}^*φ*(*m*) ^and set *f *= [*tf′ *+ 1]*_q_*. If *f *is not invertible modulo *q*, choose a new *f′ *and compute the inverse *f*^−1 ^∈ *R *of *f *modulo *q *and set *h *= [*tgf*^−1^]*q *. Let ℓ*_ω,q _*= [log*_ω _*(*q*)] + 1 and define ${\text{P}}_{\omega ,q}\left(a\right)={\left({\left[a\omega^{i}\right]}_{q}\right)}_{i=0}^{\ell_{\omega ,q^{-1}}}$. Sample $e,s\leftarrow \chi_{err}^{\ell_{\omega ,q}}$ and compute $\gamma =\left[{\text{P}}_{\omega ,q}\left(f\right)+e+hs\right]\in R_{q}^{\ell_{\omega ,q}}$ . Then we set the key pair: (pk, sk, evk) = (*h, f, γ*).

• Encryption: To encrypt *m *∈ *R_t_*, choose *e, s ← χ_err _*and then compute the ciphertext $\text{Enc}\left(m,\text{pk}\right)={\left[\left\lfloor \frac{q}{t}\right\rfloor \cdot {\left[m\right]}_{t}+e+hs\right]}_{q}\in R_{q}$.

• Decryption: Given a ciphertext ct, output $\text{Dec}(\text{ct},\text{sk})=\left\lfloor \frac{t}{q}\cdot {\left[f\cdot ct\right]}_{q}\right\rceil$ mod *t*. The inherent noise in the ciphertext is defined as the minimum value of infinite norm ||*v*||_∞ _= max*_i_*{|*v_i_*|} such that $f\cdot \text{ct}=\left\lfloor \frac{q}{t}\right\rfloor \cdot {\left[m\right]}_{t}+v{\left(\text{mod}\right)}_{q}$

• Homomorphic Evaluation: Given two ciphertexts ct and ct′, homomorphic addition is computed as $\text{c}{\text{t}}_{\text{add}}={\left[\text{ct}+\text{ct'}\right]}_{q}$

• Homomorphic Evaluation: Given two ciphertexts ct and ct′, homomorphic addition is computed as $\text{c}{\text{t}}_{\text{add}}={\left[\text{ct}+\text{ct'}\right]}_{q}$. Homomorphic multiplication is computed as $\text{c}{\text{t}}_{\text{mult}}=\text{SwitchKey}\left({\left[\left\lfloor \frac{t}{q}\text{ct}\cdot \text{ct'}\right\rceil \right]}_{q},\text{evk}\right)$ where the key switching function SwitchKey is used to transform a ciphertext decryptable under the original secret key *f *(see [19] for details).

## Our methods for private genome analysis

In this section, we describe how to encode and encrypt the genomic data for each task. Based on these methods, we propose the evaluation algorithms to compute the genomic tests on encrypted data.

### Encoding genomic data

**Figure 1:**
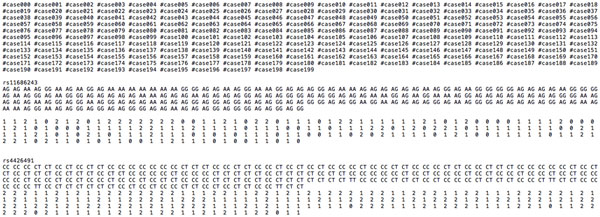
**A snapshot of the dataset for Task 1 and its encodings**.

Lauter et al. [16] presented a method to encode a person's genotype given a candidate allele associated to a specified disease. They used a binary dummy vector representation, which makes the number of ciphertexts too large. In contrast, we encode the genotypes as integers so that one can efficiently compute their sums and differences over the integers. More precisely, for a bi-allelic gene with alleles *A *and *B*, there are 3 possible Single Nucleotide Polymorphisms (SNPs) - *AA*, *AB*, *BB*, and they are encoded as follows: *AA → *2, *AB → *1, *BB → *0. Figure 1 shows the file format of the data for Task 1 and its encodings.

Now, we describe how genomic data can be encoded for DNA comparison. The first step is to curate the data using the positions in the VCF files of two participants. In other words, the server should arrange the information and make the merged list  L so that each individual can encode their genotypic information according to the list. Let ℓ(L) denote the length of the list  L. Then, for 1≤i≤ℓ(L), we define two values

$$e_{i}=\left\{\begin{array}{cc} 1 & \text{if}\;\text{po}{\text{s}}_{i}\in L\\ 0 & \text{o}\text{.w}, \end{array}\right),\;\;\;\;f_{i}=\left\{\begin{array}{cc} 0 & \text{if}\;\text{s}{\text{v}}_{i}\in \left\{\text{INS, DEL}\right\}\\ 1 & \text{o}\text{.w},. \end{array}\right)$$

The value *e_i _*defines whether the genotype at the specified locus is missing; the value *f_i _*specifies the variants compared with the reference.

Since both VCF files are aligned with the same reference genome, we don't need to compare the columns of 'REF'. To improve performance, we assume that it suffices to compare 7 SNPs between two non-reference sequences. In the following, we describe how to encode the sequences. Each SNP is represented by two bits as

A→00, G→01, C→10, T→11,

and then concatenated with each other. Next we pad with 1 at the end of the bit string so as to distinguish the *A*-strings. Finally, we pad with zeros to make it a binary string of length 15, denoted by **s***_i_*. Let **s***_i_*[*j*] denote *j*-th bit of **s***_i_*. If a person's SNV at the given locus is not known (*i.e., e_i _*= 0), then it is encoded as 0-string. For example, '*GT C*' is encoded as a bit string 01||11||10||10 ... 0, of length 15.

**Figure 2:**
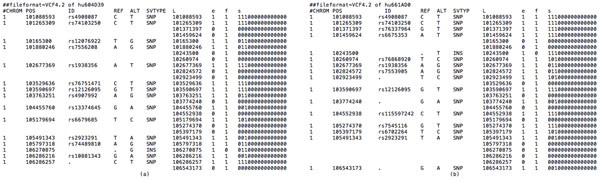
**A snapshot of the dataset for Task 2 and its encodings**. (a) hu604D39 and (b) hu661AD0.

Finally, let us consider the *i*-th genotype lengths *D_i_, $D_{i}^{\prime }$*of two participants defined as follows: when it has no variants at the given locus of the sequence, set zero as the length at the locus. If it includes a deletion compared with the reference, use the length of reference. Otherwise, we take the length of the target sequence at the current locus. In Figure 2, we illustrate the file format of the data for Task 2 and its encodings.

### Homomorphic computation of the BGV scheme

We describe how to compute the genomic algorithms described above on encrypted genetic data using the BGV scheme.

#### Task 1: GWAS on encrypted genomic data

Using the encodings that we propose for practical HE, we can homomorphically evaluate any function involving additions and multiplications, but it is not known how to perform homomorphic division of integer values. We obtain the counts using a few homomorphic additions.

Let *g_j _*be the encoded value of SNV site *j *based on the encoding method as described above. Then each person packs *g_j _*into the *j*-th slot. Let *s *be the total number of SNVs. Assuming that each ciphertext holds *ℓ *plaintext slots for *s *≤ *ℓ*, the *i*-th person encrypts the vector $\left(g_{i}^{\left(1\right)},\ldots ,g_{i}^{\left(s\right)},0\ldots ,0\right)\in {\mathbb{Z}}_{t}^{\ell }$ using batching as

$${\text{ct}}_{i}=\text{Enc}(\text{CRT}((g_{i}^{\left(1\right)},\ldots ,g_{i}^{\left(s\right)},0\ldots ,0),\text{pk}).$$

Let ct*_eval _*be a ciphertext given by the homomorphic operation

$$\text{c}{\text{t}}_{eval}=\overset{N}{\underset{i=1}{\sum }}\text{c}{\text{t}}_{i}.$$

Note that the use of batching technique enables to perform *N *aggregate operations in parallel. Next, let m = Dec(ct*_eval_*, sk) denote the decryption of the ciphertext ct*_eval _*and decode the *s *outputs from the output plaintext polynomial as follows: let m*_j _*be the constant coefficient of m mod *f_j _*for 1 *≤ j ≤ s*. That is, we have

mj =letm modfj= ∑i=1Ngi(j).

Thus the MAF of SNV *j *in the group is computed as

$$\frac{\text{min}\left\{{\text{m}}_{j},2N-{\text{m}}_{j}\right\}}{2N}.$$

For the homomorphic evaluation of *χ*^2 ^test, each group performs aggregations over ciphertexts as shown in (1). Let ct*_case _*and ct*_cont _*denote the ciphertexts by the evaluations in the case and control groups, respectively. Then one can compute two ciphertexts by the homomorphic operations

$$\text{c}{\text{t}}^{+}\overset{let}{=}\text{c}{\text{t}}_{case}+\text{c}{\text{t}}_{cont},\;\;\text{c}{\text{t}}^{-}\overset{let}{=}\text{c}{\text{t}}_{case}-\text{c}{\text{t}}_{cont}.$$

The plaintext polynomial from ct^+ ^can be decoded as the plaintext slots which have $\left(n_{A}^{\left(j\right)}+{n^{\prime }}_{A}^{\left(j\right)}\right)$ at the *j*-th slot. In other words, we have

$$\text{Dec}({\text{ct}}^{+},\text{sk})\;\mathrm{mod}\;f_{j}=\sum_{i=1}^{N}\left(g_{i}^{\left(j\right)}-{g^{\prime }}_{i}^{\left(j\right)}\right)=(n_{A}^{\left(j\right)}-{n^{\prime }}_{A}^{\left(j\right)}).$$

Similarly, the plaintext polynomial from ct^− ^is decoded as the plaintext slots which has the value congruent to $\left(n_{A}-n_{A}^{\prime }\right)$ in the interval [0,t)∩ℤ. Thus, if the output value is larger than $\frac{t}{2}$, then subtract *t *from it; that is, we have

$$\text{Dec}({\text{ct}}^{+},\text{sk})\;\mathrm{mod}\;f_{j}=\sum_{i=1}^{N}\left(g_{i}^{\left(j\right)}-{g^{\prime }}_{i}^{\left(j\right)}\right)=(n_{A}^{\left(j\right)}-{n^{\prime }}_{A}^{\left(j\right)}).$$

#### Task 2: secure DNA sequence comparison

We represent sequence comparison algorithms as binary circuits and then evaluate them over encrypted data. We use the native plaintext space of binary polynomials (*i.e., *$R_{2}={\mathbb{Z}}_{2}\left[x\right]/\left(\Phi_{m}\left(x\right)\right)$), and denote XOR and AND as ⊕ and ∧, respectively. For simplicity, you may consider the plaintext space ${\mathbb{Z}}_{2}^{\ell }$ supporting batching operation with ℓ slots.

For the homomorphic evaluation of Hamming distance, the genomic data of two participants, denoted by $\left(e_{i},f_{i},s_{i}\right)$ and $\left(e_{i}^{\prime },f_{i}^{\prime },s_{i}^{\prime }\right)$, are encrypted bit-wise. For example, the encryptions of *e_i_*'s are in the form of

$$\begin{array}{c} \text{Enc}\;\text{(CRT(}e_{1},\ldots ,e_{\ell }\text{)},\text{pk)},\\ \text{Enc}\;\left(\text{CRT(}e_{\ell +1},\ldots ,e_{2\ell }\text{)},\text{pk}\right)\;,\ldots ,\\ \text{Enc}\;\left(\text{CRT(}e_{\left\lfloor \ell \left(L\right)/\ell \right\rfloor \cdot \ell +1},\ldots ,e_{\ell \left(L\right)},0,\ldots 0\text{),}\;\text{pk}\right). \end{array}$$

This allows to compute the same function on ℓ inputs at the price of one computation. Then one can evaluate the following binary circuit over encryption:

$$\left(\text{E}\left({\text{s}}_{i},{\text{s'}}_{i}\right)\wedge \left(e_{i}⊕{e^{\prime }}_{i}⊕1\right)⊕1\right)\wedge f_{i}\wedge f_{i}^{\prime }$$

where $\text{E(}{\text{s}}_{i},{\text{s'}}_{i}\text{)}=\wedge_{j=1}^{15}\left({\text{s}}_{i}\left[j\right]⊕{\text{s'}}_{i}\left[j\right]⊕1\right)$ has 1 if and only if ${\text{s}}_{i},{\text{s'}}_{i}$ are the same. After homomorphic computations, the output can be decrypted with the secret key. The plaintext polynomial has the Hamming distance result of SNV site *i *at the *i*-th slot, so we need only aggregate them.

Now, we consider the comparison binary circuit (described in [17]) for the secure computation of the approximate Edit distance. We express an unsigned *μ*-bit integer *x *in its binary representation and denote the *j*-th coordinate of *x *by *x*[*j*] (*i.e*., $x=\sum_{j=1}^{\mu }x\left[j\right]\;2^{j-1},x\left[j\right]\in \left\{0,1\right\}$). For two *μ*-bit integers *x *and *y*, the comparison circuit is defined by

$$\text{C}\left(x,y\right)=\left\{\begin{array}{cc} 1 & \text{if}\;x<y,\\ 0 & \text{o}.\text{w}., \end{array}\right)$$

and this is written recursively as C(*x*; *y*) := *c_μ _*where

$$c_{j}=\left(\left(x\left[j\right]⊕1\right)\wedge y\left[j\right]\right)⊕\left(\left(x\left[j\right]⊕1⊕y\left[j\right]\right)\wedge c_{j-1}\right)$$

for *j *≥ 2 with an initial value $c_{1}=\left(x\left[1\right]⊕1\right)\wedge y\left[1\right]$. Then the *j*-th bit of maximum value between two inputs is defined as follows:

$$\begin{array}{c} \text{max}\left\{x,y\right\}\left[j\right]=\left(\left(1⊕\text{C(}x,y\text{)}\right)\wedge x\left[j\right]\right)⊕\left(c\left(x,y\right)\wedge y\left[j\right]\right)\\ =x\left[j\right]⊕\left(\text{C(}x,y\text{)}\wedge \left(x\left[j\right]⊕y\left[j\right]\right)\right). \end{array}$$

For the bit-sliced implementation, all the lengths are also expressed in a binary representation and we denote the maximum length of SNPs by *μ*. It follows from the primitive circuits that we can evaluate the circuits homomorphically:

$$\left(E\left({\text{s}}_{i},{\text{s'}}_{i}\right)\wedge \left(f_{i}⊕{f^{\prime }}_{i}⊕1\right)⊕1\right)\wedge \text{max}\left\{D_{i},\overset{\prime }{\underset{i}{D}}\right\}\left[j\right].$$

Finally, one can decrypt the results and decode ℓ(L) values from the output plaintext polynomials. More precisely, let $\ell_{i,j}$ be the value at *i*-th slot which corresponds to the *j*-th bit. We see that $\sum_{j=1}^{\mu }\ell_{i,j}\cdot 2^{j-1}$ is the approximate Edit distance of SNV site *i*, hence we need only perform aggregation operations over them.

### Homomorphic computation of the YASHE scheme

We explain how to evaluate the genomic algorithms homomorphically using the YASHE scheme.

#### Task 1: GWAS on encrypted genomic data

Lauter et al. [13] introduced a method how to pack *m *bits *b*_0_, ..., *b*_*m*−1 _into a single ciphertext that encodes the polynomial $b\left(x\right)=\sum_{i=0}^{m-1}b_{i}x^{i}$. We note that polynomial addition corresponds to simple component-wise addition of the vectors. Since a case-control study requires only additions, this method can be used for our case. When using a ring polynomial *x^n ^*+1 with a power-of-two *n*, we can embed data of $n^{\prime }\;\overset{let}{=}\;\left\lfloor \frac{n}{s}\right\rfloor$ persons into a single plaintext polynomial. Namely, one can encrypt the polynomial

$$\begin{array}{c} \text{pm}\left(g_{1}=\left(g_{1}^{\left(1\right)},\ldots ,g_{1}^{\left(s\right)}\right),\;\ldots \;,g_{n^{\prime }}=\left(g_{n^{\prime }}^{\left(1\right)},\ldots ,g_{n^{\prime }}^{\left(s\right)}\right)\right)\\ \overset{let}{=}\;\overset{n^{\prime }}{\underset{i=1}{\sum }}\overset{s-1}{\underset{j=0}{\sum }}g_{i}^{\left(j\right)}x^{j+s\cdot \left(i-1\right)}. \end{array}$$

The simple aggregation operations are performed over packed ciphertexts. Now, let

$$\text{m}=\overset{n^{\prime }s-1}{\underset{j=0}{\sum }}\;{\text{m}}_{j}x^{j}\in R_{t}$$

denote the decryption result of the evaluated ciphertext. Then, for 1 ≤ *j *≤ *s*, one can aggregate *n^' ^*data from the output plaintext polynomial by computing

$$m_{j}\leftarrow \overset{n^{\prime }-1}{\underset{i=0}{\sum }}\;{\text{m}}_{j+is},$$

which is the allele counts of *A *at the SNV site *j*. Notice that if $n^{\prime }=1$, then we don't need to do the above operations. Hence, the MAF of the SNV *j *in the group is computed as

$$\frac{\text{min}\left\{{\text{m}}_{j},2N-{\text{m}}_{j}\right\}}{2N}.$$

Similarly, let ct^+ ^and ct^− ^denote the ciphertexts computed by the homomorphic additions and subtractions after simple aggregations. As we have demonstrated, we need additional aggregation processes after decryptions. Let

$${\text{m}}^{+}=\overset{n^{\prime }\;s-1}{\underset{j=0}{\sum }}m_{j}^{+}x^{{}_{j}},\;m^{-}=\overset{n^{\prime }\;s-1}{\underset{j=0}{\sum }}m_{j}^{-}x^{{}_{j}}$$

denote the decryption polynomials of ct^+ ^and ct^−^, respectively. Then, for 1 ≤ *j *≤ *s*, one can obtain the allele counts by computing as

$$n_{A}^{\left(j\right)}+n_{A}^{{}^{\prime }\left(j\right)}=\overset{n^{\prime }-1}{\underset{i=0}{\sum }}\;{\text{m}}_{j+is}^{+},\;n_{A}^{\left(j\right)}-n_{A}^{{}^{\prime }\left(j\right)}={\left[\overset{n^{\prime }-1}{\underset{i=0}{\sum }}\;{\text{m}}_{j+is}^{-}\right]}_{t}.$$

#### Task 2: secure DNA sequence comparison

Since polynomial multiplication does not correspond to component-wise multiplication of the vectors, we have to consider another packing method instead of [13]. Let us consider the polynomial-CRT packing method. The *m*-th cyclotomic polynomial $\Phi_{m}\left(x\right)$ factors modulo 2 into a product of the same irreducible factors (*i.e*., $\Phi_{m}\left(x\right)=x^{n}+1={\left(x+1\right)}^{n}$ mod 2); so we cannot apply batching technique with these parameters. We can instead do that if taking a prime *t *(not 2) such that the polynomial splits into the distinct factors modulo *t*, but the use of a different message space leads to change our primitive circuits.

As noted in [27], we see that for x,y∈{0,1}, the following properties hold: $x⊕y={\left(x-y\right)}^{2}$ and x∧y=x⋅y where − and · are arithmetic operations over integers. From these observations, we can amend the evaluation circuit for the Hamming distance as follows:

$$\left(\text{E}(s_{i},{s^{\prime }}_{i})\cdot \left({\left(e_{i}-{e^{\prime }}_{i}\right)}^{2}-1\right)+1\right)\cdot f_{i}\cdot {f^{\prime }}_{i}$$

where $\text{E}\left({\text{s}}_{i},{{\text{s}}^{\prime }}_{i}\right)=\overset{15}{\underset{j=1}{\prod }}\left(1-{\left({\text{s}}_{i}\left[j\right]-{{\text{s}}^{\prime }}_{i}\left[j\right]\right)}^{2}\right)$.

We note that for μ-bit integer *x *and *y*, the comparison circuit C(*x*; *y*) = *c_μ _*can be expressed as

$$c_{j}=\left(1-x\left[j\right]\right)\cdot y\left[j\right]+\left(1-{\left(x\left[j\right]-y\left[j\right]\right)}^{2}\right)\cdot c_{j-1}.$$

for *j *≥ 2 with $c_{1}=\left(1-x\left[1\right]\right)\cdot y\left[1\right]$ Since it is available to compute on large integer inputs, the maximum value is defined by

$$\begin{array}{c} \text{max}\left\{x,y\right\}=\left(1-\text{C}\left(x,y\right)\right)\cdot x+\text{C}\left(x,y\right)\cdot y\\ =x+\text{C}\left(x,y\right)\cdot \left(y-x\right). \end{array}$$

Using these circuits, we compute the ciphertext given by the homomorphic operations

$$\left(1+\text{E}\left({\text{s}}_{i},{\text{s'}}_{i}\right)\cdot \left({\left(f_{i}-{f^{\prime }}_{i}\right)}^{2}-1\right)\right)\cdot \text{max}\left\{D_{i},{D^{\prime }}_{i}\right\}.$$

Then we get the encryptions of the approximate Edit distance result of SNV *i*.

## Results and discussion

In this section, we explain how to set the parameters for homomorphic evaluations and present our experimental results. We used BGV scheme with Shoup-Halevi's HE library [28] (called HELib). HELib is written in C++ and based on the arithmetic library NTL [29] over GMP. Our experiments with BGV were performed on a Linux machine with an Intel Xeon 2.67 GHz processor. We also implemented YASHE scheme with ARITH library in C. The measurements were done in an Intel Core 3.60GHz, running 64-bit Windows 7.

The dataset used for Task 1 consists of 200 case group (constructed from 200 participants from PGP) and 200 control group (simulated based on the haplotypes of 174 participants from CEU population of apMap Project). The dataset for Task 2 consists of two individual genomes randomly selected from PGP.

### Theoretical comparison between BGV and YASHE

BGV scheme has a chain of ciphertext moduli by a set of primes of roughly the same size, *p*_0_, ⋯, *p*_*L*−1_, that is, the *i*-th modulus *q_i _*is defined as $q_{i}=\prod_{k=0}^{i}pk$. For simplicity, assume that *p *is the approximate size of the *p_i_*s. Given the lattice dimension n=φ(m), the plaintext modulus *t*, and the Hamming weight *h *of the secret key, it follows from Theorem 3 in [27] that the depth of a classical homomorphic multiplication is

$$d_{n,t}\approx \left\lceil \frac{{\text{log}}_{2}\left(h\cdot n\cdot t^{4}\right)}{2{\text{log}}_{2}\left(p\right)}\right\rceil \approx \left\lceil \frac{{\text{log}}_{2}\left(h\cdot n\cdot t^{4}\right)}{36}\right\rceil ,$$

so the total number of modulus switching operations during the **M**-levels of multiplications is about **M**·*d_n,t_*. Since we first should do one modulus switching to the initial ciphertext before homomorphic computation, we see that *L *= *M *· *d_n,t _*+ 2. Thus we can approximate the size of the ciphertext modulus *q*_BGV _in the BGV scheme (from C.3 in [18]) as follows:

$$\begin{array}{c} {\text{log}}_{2}q_{\text{BGV}}\approx 24+\frac{3}{2}{\text{log}}_{2}n+\left(L-2\right)\cdot \left(11+\frac{1}{2}{\text{log}}_{2}n\right)\\ <\left(L+1\right)\cdot \left(11+\frac{1}{2}{\text{log}}_{2}n\right) \end{array}$$

Since a fresh ciphertext in BGV consists of a pair of polynomials over *R*_*qL*−1_, the size of ciphertext from the above inequality is about

$$|\text{c}{\text{t}}_{\text{BGV}}|\;\approx 2n\cdot {\text{log}}_{2}q_{\text{BGV}}\approx 2n\left(L+1\right)\cdot \left(11+\frac{1}{2}{\text{log}}_{2}n\right)$$

Similarly, [19, Lemma 9] provides a theoretical upper bound on the noise growth after M multiplicative levels for YASHE as (*nt*)^2(M*−*1) ^*· *(12*n*^2^*tσ*ℓ_*ω,q*_*ω*M) when taking *B *= 6*σ *as the coefficient bound of error polynomials. It should be less than the ratio of *q*_YASHE _to *t *so that the decryption procedure works; we should select a ciphertext modulus *q*_YASHE _so as to satisfy

$$\begin{array}{c} {\text{log}}_{2}q_{\text{YASHE}}\approx 2\text{M}\cdot {\text{log}}_{2}nt+{\text{log}}_{2}\left(12\sigma \ell_{\omega ,\;q}\omega \text{M}\right)\\ \geq 2\text{M}\cdot \left({\text{log}}_{2}nt\right) \end{array}$$

Since a ciphertext consists of only a single ring element, the size is about

$$|\text{c}{\text{t}}_{\text{YASHE}}|\;\approx n\cdot {\text{log}}_{2}q_{\text{YASHE}}\approx 2n\text{M}\cdot \left({\text{log}}_{2}nt\right).$$

We summarize the above results in Table 2.

**Table 2:** The theoretical sizes of ciphertext modulus and a ciphertext

	BGV	YASHE
Log_2 _*q*	$\left(\text{M}\cdot \frac{{\text{log}}_{2}\left(h\cdot n\cdot t^{4}\right)}{36}+3\right)\cdot \left(11+\frac{1}{2}\;{\text{log}}_{2}n\right)$	2M · log_2 _*nt*

|ct|	$2n\left(\text{M}\cdot \frac{{\text{log}}_{2}\left(h\cdot n\cdot t^{4}\right)}{36}+3\right)\cdot \left(11+\frac{1}{2}\;{\text{log}}_{2}n\right)$	2*n*M · log_2 _*nt*

Note that it is difficult to compare these two schemes because their parameters depend on at least 4 variables: the plaintext modulus, *t*, the dimension, *n*, the Hamming weight, *h*, and the number of multaplicative levels to be evaluated, M. However we observe that, in the case that log_2 _*n ≈ *14 and *h *= 64, we have:

$$\begin{array}{c} {\text{log}}_{2}q_{\text{YASHE}}-{\text{log}}_{2}q_{\text{BGV}}\\ \approx 2\text{M}\cdot \left({\text{log}}_{2}nt\right)-\left(M\cdot d_{n,t}+3\right)\cdot \left(11+\frac{1}{2}{\text{log}}_{2}n\right)\\ \approx 2\text{M}\cdot \left(14+{\text{log}}_{2}t\right)-\left(\text{M}\cdot d_{n,t}+3\right)\cdot 18\\ =2\text{M}\cdot \left(14+{\text{log}}_{2}t-9\cdot d_{n,t}\right)-54\\ \approx 2\text{M}\left(14+{\text{log}}_{2}t-9\cdot \left(\frac{20+4\;{\text{log}}_{2}t}{36}+\eta \right)\right)-54\\ =18\text{M}\left(1-\eta \right)-54\;\text{for}\;\text{some}\;0\leq \eta <1. \end{array}$$

Hence, if M is large, we can use a smaller ciphertext modulus to evaluate M-levels of multiplications with BGV in comparison to YASHE; however, the YASHE scheme has smaller ciphertexts than BGV. This follows from the fact that

$$\begin{array}{c} \left|\text{c}{\text{t}}_{\text{BGV}}|-|\text{c}{\text{t}}_{\text{YASHE}}\right|\\ \approx 2\left(\text{M}\cdot d_{n,t}+3\right)\cdot \left(11+\frac{1}{2}{\text{log}}_{2}n\right)-2\text{M}\cdot \left({\text{log}}_{2}nt\right)\\ \approx 2\text{M}\cdot \left(18\cdot d_{n,t}-14-{\text{log}}_{2}t\right)+108\\ \approx 2\text{M}\cdot \left({\text{log}}_{2}t+18\eta -4\right)+108 \end{array}$$

For some 0 ≤ η < 1; if log_2 _*t *≥ 4, then log_2 _*t *+ 18η - 4 ≥ 0; otherwise, we have *d*_*n*,*t *_= 1 and so $18\cdot d_{n,t}-14-{\text{log}}_{2}t>0$.

Let us contrast the complexity of homomorphic multiplication operations for the two schemes. One of the new optimizations for BGV is to convert polynomials between coefficient and evaluation representations. Most of the homomorphic operations are performed in the more efficient evaluation representation, but it sometimes requires coefficient representation. Note that these conversions take the most time in execution. In more detail, at the *l*-th level of this scheme, the key switching procedure requires  O(*l*) Fast Fourier Transforms (FFTs) and the modulus switching operation requires (*l *+ 1) FFTs. Since HElib uses the Bluestein FFT algorithm [30] (with run-time complexity of  O(*n *log *n*)), this yields an overall complexity of  O(*ln *log *n*) for a multiplication of ciphertexts.

For the polynomial multiplication in the base ring *R_q _*= ℤ*_q_*[*x*]/(*x^n ^*+ 1), we implemented the FFT algorithm by Nussbaumer [31] based on recursive negacyclic convolutions (with run-time complexity $\frac{9}{2}n\;\text{log}n\;\text{log}\;\text{log}n+O\left(n\text{log}n\right)$ of arithmetic operations in ℤ*_q_*). The homomorphic multiplication in YASHE includes a costly key switching operation which is an inner product on $R_{q}^{\ell \omega ,q}$, hence we obtain a total cost of $\ell_{\omega ,q}\cdot \left(\frac{9}{2}n\;\text{log}\;n\;\text{log}\;\text{log}\;n+O\left(n\text{log}n\right)\right)$ operations for a ciphertext multiplication. Therefore, BGV is expected to be faster than YASHE for a ciphertext multiplication if we take similar parameters with *q *and *n*.

### How to set parameters

The security of BGV relies on the hardness of the RLWE assumption. Similarly, YASHE is provably secure in the sense of IND-CPA under the RLWE assumption and DSPR assumption. The main difference between the schemes is that BGV uses an odd integer *m *while YASHE chooses *m *to be a power-of-two with a prime integer *q *such that *q ≡ *1 (mod *m*). In [23], it was shown that the hardness of RLWE with the cyclotomic polynomial Φ*_m_*(*x*) = *x*^*ϕ*(*m*) ^+ 1 can be established by a quantum reduction to shortest vector problems in ideal lattices. This means that YASHE is believed to be secure as long as the lattice problems are hard to solve.

#### Parameters of the BGV scheme

To homomorphically evaluate the algorithms for Task 1, we first choose sufficiently large *t *so that no reductions modulo *t *occurs in the plaintext slots. For example, we take *t *as the smallest power-of-two which satisfies the following inequalities:

$$n_{A}^{\left(j\right)}=\overset{200}{\underset{i=1}{\sum }}g_{i}^{\left(j\right)}\leq \overset{200}{\underset{i=1}{\sum }}2=400<t$$

since the total number of people in the same group is *N *= 200. So it suffices to take *t *= 2^9 ^for privately computing the minor allele counts. In the case of *χ*^2 ^test, we have

$$n_{A}^{\left(j\right)}+{n^{\prime }}_{A}^{\left(j\right)}=\sum_{i=1}^{200}g_{i}^{\left(j\right)}+\sum_{i=1}^{200}{g^{\prime }}_{i}^{\left(j\right)}\leq 2\sum_{i=1}^{200}2=800<t,$$

thus we set the parameter *t *= 2^10^. For the second task, we used *t *= 2 to evaluate binary circuits.

Now, we derive a lower-bound on *ϕ*(*m*) such that

(2) $$\phi \left(m\right)\geq \frac{\left(L\left(\text{log}m+23\right)-8.5\right)\cdot \left(\lambda +110\right)}{7.2}.$$

from the security analysis of [18] based on Lindner and Peikert's method [32]. For the efficiency of the implementation, we choose the smallest integer *m *so as to satisfy Inequality (2) and pack the message into plaintext slots as many as possible. Next, we define a ladder of moduli to make the correct decryption after computation with *L *levels (see [18] for details). Finally, we consider the discrete Gaussian distribution *χ_err _*= *D*_ℤ,*σ *_with mean 0 and standard deviation *σ *= 3.2 over the integers to sample random error polynomials.

#### Parameters of the YASHE scheme

As discussed before, *t *= 2^10 ^will suffice to compute the MAFs and *χ*^2 ^statistic. For the second task, we look for the parameter *t *≠ 2 which maximizes the number of slots we can handle in one go. We fix the word *ω *= 2^128 ^for the evaluation key and the standard deviation *σ *= 8 for the error distribution *χ_err_*.

Since we can estimate the size of noise during homomorphic operations, we get the lower bound on *q *to ensure the correctness. We also have maximal values of *q *to ensure the desired security using the results of [33], so that we can have more loose bound than that from LP's method. Then we set *m *as a power-of-two to get a non-trivial interval for *q *and then select a smallest *q *in this interval.

### Implementation results

We present the parameter setting and performance results for secure genome analysis in Table 3 and 4. All the parameters provide 80-bit security level. We give the plaintext modulus *t*, the size of the ciphertext modulus *q*, the lattice dimension *n *= *ϕ*(*m*), and the number of plaintext slots ℓ. We also give the circuit depth *L *so that HE scheme can correctly evaluate such a computation on encrypted data. In particular, it can be considered as the number of ciphertext moduli in the BGV scheme. We consider the ciphertext size in kBytes for a set of parameters. The last columns give the timings for the key generation, encryption, evaluation and decryption.

**Table 3:** Implementation results of Task 1 using BGV and YASHE

		*s*	*t*	log_2 _*q*	*n*	ℓ	*L*	|ct|	KeyGen	Encrypt	Eval	Decrypt
BGV	**MAF**	311 610	2^9^	60 61	5292 8190	378 630	3	78 kB 122 kB	6.92*s* 10.28*s*	11.90*s* 14.85*s*	**29.99 ms** **33.36 ms**	290.06 *ms* 690.23 *ms*

	*χ***^2^**	311 610	2^10^	60 61	5292 8190	378 630	3	78 kB 122 kB	6.35*s* 12.27*s*	11.61*s* 15.13*s*	**30.05 ms** **38.17 ms**	560.10 *ms* 720.33 *ms*

YASHE	**MAF**	311 610	2^10^	48	1024	1024	0	6 kB	0.01*s* 0.04*s*	1.63*s* 4.10*s*	**5.74 ms 16.98 ms**	33.71 *ms* 16.78 *ms*

	*χ***^2^**	311 610							0.01*s* 0.04*s*	1.61*s* 4.12*s*	**5.99 ms** **17.20 ms**	16.73 *ms* 17.01 *ms*

**Table 4:** Implementation results of Task 2 using BGV and YASHE

		Size	*t*	log_2 _*q*	*n*	ℓ	*L*	|ct|	KeyGen	Encrypt	Eval	Decrypt
BGV	**Hamming**	5K 10K	2	132	8190	630	7	264 kB	2.53*s*	12.65*s* 24.90*s*	**15.39s** **29.39s**	0.64*s* 1.29*s*

	**Edit**	5K 10K		150			8	300 kB	3.41*s*	16.98*s* 33.34*s*	**40.86s** **76.08s**	2.97*s* 5.81*s*

YASHE	**Hamming**	5K 10K	8191	384	8192	4096	6	384 kB	130.59*s*	29.70*s* 58.82*s*	**68.31s** **134.87s**	2.67*s* 5.04*s*

	**Edit**	5K 10K								58.46*s* 116.61*s*	**110.18s** **245.04s**	2.66*s* 5.07*s*

#### Performance results of Task 1

In Table 3 the top four rows refer to the results using BGV, and the bottom four rows refer to results using YASHE for computing the MAFs and *χ***^2 ^**statistic in case-control groups. Note that the number of slots means that how many messages we can pack into one single ciphertext. When using YASHE, we can evaluate simultaneously by embedding the data into the coefficients of plaintext polynomial; the maximal degree of plaintext polynomial in this case is considered to be the number of slots.

In practice, we need to apply one more modulus-switching during homomorphic additions for the BGV scheme, so the total number of ciphertext moduli is *L *= 1 + 2 = 3. On the contrary, *L *means the levels of multiplications in YASHE (without taking into account the additions). In other words, when evaluating a polynomial of degree *d *on encrypted data, we have *L ≈ *log *d *levels of multiplications by computing in a binary tree way. Thus, *L *= 0 suffices to support such homomorphic additions in Task 1. Thus we don't need to generate the evaluation key, which enables to take less time for key generation than BGV. Moreover, the evaluation performance of YASHE is much better since BGV requires a costly modulus switching operations even for computing simple homomorphic additions.

#### Performance results of Task 2

Table 4 presents the parameter setting and performance results for secure DNA sequence comparison using BGV and YASHE. We evaluated the performance with the input data of different sizes 5*K *and 10*K*. We implemented the comparison circuit with the same method as described in [17, Lemma 1] in order to reduce the circuit depth over encryption.

As discussed before, given the parameter *L*, we obtain the approximate size of ciphertext modulus as log_2 _*q ≈ *43 + 18 *· *(*L − *2) for BGV when using *t *= 2 and *R *= ℤ[*x*]/(Φ_8191_(*x*)). Since it should support *L *= 7 or 8 to correctly evaluate genomic algorithms of Task 2, we use the modulus *q *around 130 to 150. On the other hand, the size of the parameter *q *in YASHE should be strictly larger than 2*L *log_2 _(*nt*) *≈ *52*L *with *t *= 2^9 ^and *R *= ℤ[*x*]/(*x*^8192 ^+ 1). So we used a 384-bit prime *q *such that *q ≡ *1 (mod 2^14^).

In the implementation of YASHE scheme, computing the inverse of *f *modulo *q *turns out to be the most-time consuming part of the key-generation, which runs in around 128.34 seconds(s). In total, it takes about 130.59s to generate the public key, secret key and evaluation keys, while the key generation of the BGV scheme takes about 3.41s in order to support 8 levels.

There is also quite a big gap between the two schemes in timings for a multiplication of ciphertexts: BGV takes around 0.07s, while YASHE takes around 1.75s (including the key switching step) under the parameter settings used in Task 2. For the efficiency of the YASHE scheme, we might avoid a costly key switching step during the homomorphic multiplication; however, it supports a limited number of homomorphic multiplications without the key switching step. This follows since the noise grows exponentially with the multiplicative depth through such consecutive operations. One alternative is to use a *hybrid approach*, in which we leave out key switching in certain places but do it in others using the evaluation key with a power of the secret key so that one can keep the ciphertext noise small for correct decryption. As a result, polynomial multiplication modulo *x^n ^*+ 1 takes about 0.64s, but it is still slower than that in BGV. As expected, BGV is faster than YASHE to evaluate the genomic algorithms for DNA sequence comparison.

## Conclusions

In this paper, we discussed how to privately perform genomic tests on encrypted genome data using homomorphic encryption. In addition to the efficient implementations of BGV and YASHE, we compared two schemes both theoretically and practically. We found that there is a trade-off between the security and performance. YASHE uses a power-of-two dimension *n *which defines the 2*n*-th cyclotomic polynomial; this is a good choice for providing strong security, but it requires larger parameters to ensure correctness than BGV, and the homomorphic multiplication in YASHE is slower than that in BGV. Therefore, the performance numbers for BGV are better than YASHE when homomorphically evaluating deep circuits (like the Hamming distance algorithm or approximate Edit distance algorithm). On the other hand, it is more efficient to use the YASHE scheme for a low-degree computation, such as minor allele frequencies or *χ*^2 ^test statistic in a case-control study.

## Competing interests

The authors declare that they have no competing interests.

## Authors' contributions

MK and KL designed the baseline methods. MK drafted the manuscript and conducted the experiment for the competition. KL guided the experimental design and provided detailed edits.

## Declarations

Publication funding for this supplement was supported by iDASH U54HL108460, iDASH linked R01HG007078 (Indiana University), NHGRI K99HG008175 and NLM R00LM011392.
